# The genes *slyA, STM3120* and *htrA* are required for the anticancer ability of VNP20009

**DOI:** 10.18632/oncotarget.13217

**Published:** 2016-11-08

**Authors:** Xiaoxin Zhang, Qiaoqiao Xu, Lirun Yang, Yueyang Lai, Zhuangzhuang Zhang, Chao Han, Chizhou Jiang, Jiahuang Li, Yixin Shi, Zi-Chun Hua

**Affiliations:** ^1^ The State Key Laboratory of Pharmaceutical Biotechnology, School of Life Science and School of Stomatology, Affiliated Stomatological Hospital, Nanjing University, Nanjing, 210023, Jiangsu, China; ^2^ The School of Life Sciences and the Center for Infectious Diseases and Vaccinology at the Biodesign Institute, Arizona State University, Tempe, Arizona, 85287-4501, USA; ^3^ Changzhou High-Tech Research Institute of Nanjing University and Jiangsu TargetPharma Laboratories Inc., Changzhou, 213164, Jiangsu, China; ^4^ The State Key Lab of Natural Medicines, China Pharmaceutical University, Nanjing 210017, China; ^5^ The State Key Laboratory of Bioelectronics, Southeast University, Nanjing 210008, China

**Keywords:** VNP20009, cancer, bacteria, tumor-targeting, immune response

## Abstract

VNP20009 is a very effective anti-cancer agent and can specifically target tumors and inhibit tumor growth. It was assumed that the tumor targeting ability of VNP20009 correlated to its anticancer capacity. However, our observation contradicted to this assumption. Three VNP20009 mutant strains (*ΔslyA*, *ΔSTM3120* and *ΔhtrA*) with reduced fitness in normal tissues and unchanged fitness in tumors partially or completely lost their anti-cancer capacities. The genes *slyA*, *STM3120* and *htrA* were required for survival within macrophages and were indispensable for tumor microenvironment remodeling by VNP20009. The infiltration of immune cells occurred less in the tumors of mice infected with the mutant strains. In addition, the mRNA levels of TNF-α and IL-1β were significantly decreased in the tumors of mice treated with the mutant strains. Our results indicate that the immune responses elicited by bacteria rather than the bacterial titer in tumors play a “decisive” role in VNP20009-mediated bacterial cancer therapy, which provides a novel perspective for the underlying mechanism of bacterial cancer therapy.

## INTRODUCTION

One of the major problems in the current cancer therapies is poor tumor targeting specificity. To address this limitation, bacterial cancer therapy was developed. *Salmonella enterica* serovar Typhimurium was reported to preferentially accumulate in tumors and inhibit tumor growth [1]. However, wild-type *S. enterica* is highly pathogenic for humans. As such, avirulent *S. enterica* A1-R and VNP20009 were developed for therapeutic applications. A1-R is auxotrophic for leucine and arginine. And it has been proven to eradicate primary and metastatic tumors in nude mouse models of prostate, breast, ovarian and pancreatic cancer, etc. [2–7]. VNP20009, with double mutations in *msbB* and *purI*, is another attenuated *S. enterica* and has been proven safe in phase I clinical trials. Furthermore, VNP20009 was most wildly used to deliver anticancer agents and carry shRNA-expressing plasmids [8–12]. However, VNP20009 failed to target tumors and inhibit tumor growth in the phase I study. Therefore, additional studies are required to study the factors affecting tumor colonization and anti-tumor effects. To investigate this, a high-throughput method was used to screen for *S. enterica* promoters and genes that expressed in tumors [13, 14]. Furthermore, *in vivo* gene mutation assay showed that SPI-2, *invA* and flagella (fla) are essential for the anticancer ability of *S. enterica* [15, 16].

Macrophages play a pivotal role in bacterial clearance during an infection. After being phagocytosed by macrophages, *S. enterica* modify the vacuoles where they reside and subsequently disseminate throughout the body [17]. Fileds and his colleagues showed that the mutants that cannot survive within macrophages are avirulent [18]. As such, the capacity of *S. enterica* to cause systematic infection relies on its ability to survive and replicate within macrophages. However, the anticancer ability of the mutants lacking macrophage survival ability has not been well studied.

In this study, we knocked out three genes related to VNP20009 survival within macrophages. The gene *slyA* and *STM3120* are transcriptional regulators and HtrA is a stress protein for degrading the damaged and misfolded proteins. These genes perform different functions for bacterial survival within macrophages. Although Δ*slyA*, Δ*STM3120* and Δ*htrA* displayed reduced fitness in spleen and unchanged fitness in tumors, their anticancer capacities were partially or completely abrogated. Therefore, we further examined the immune responses by comparing immune cells and cytokines in tumor infected by VNP20009 with the mutants, attempting to elucidate the mechanism of the mutants with reduced anti-cancer capacity.

## RESULTS

### *In vitro* characterization of *ΔslyA*, *ΔSTM3120* and *ΔhtrA*

**Figure 1:**
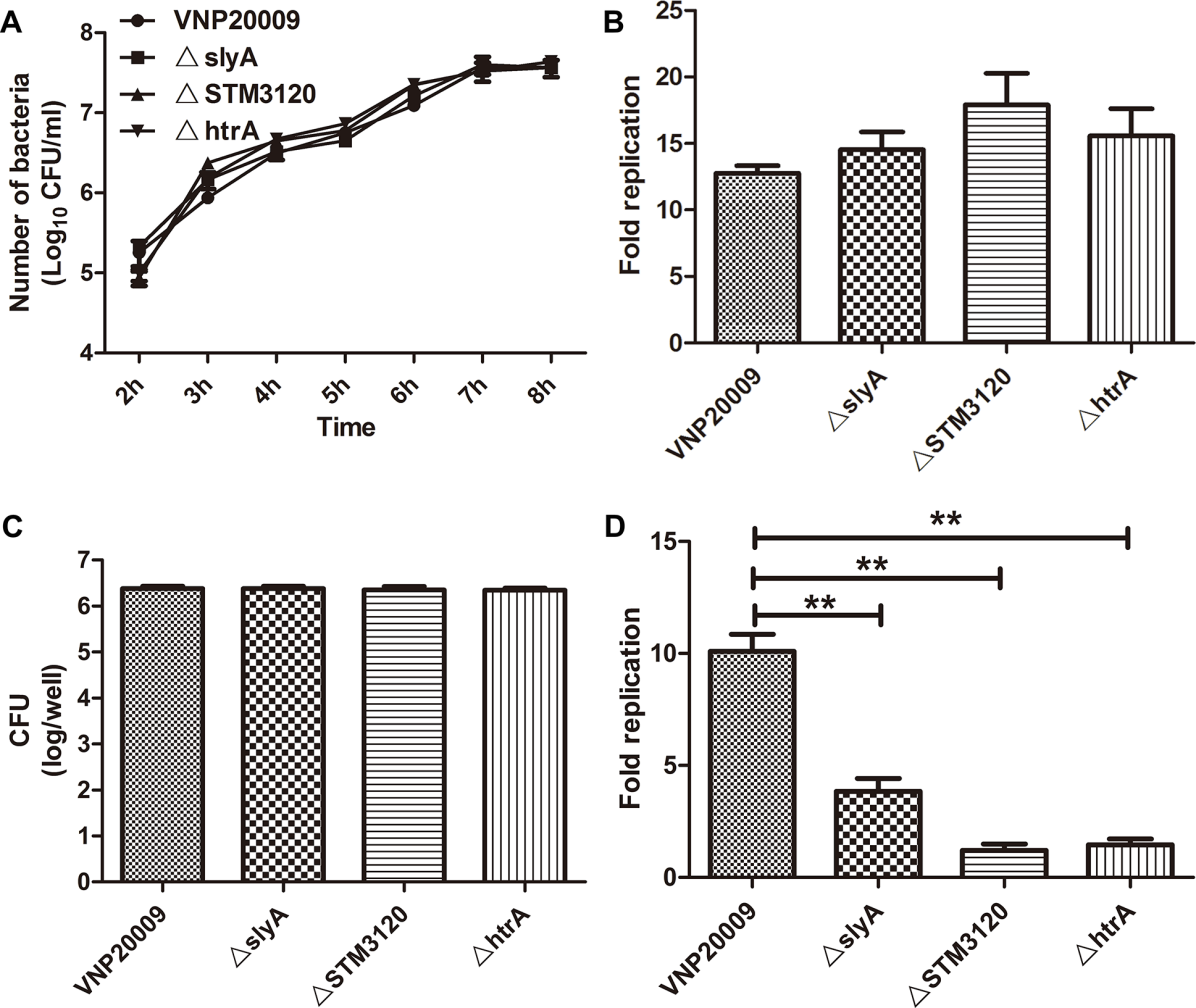
Characterization of VNP20009 and its mutants (**A**) Growth rates of VNP20009 and its mutants *in vitro*. Date are shown in mean ± SEM. (**B**) Replication rates of VNP20009 and its mutants in B16F10 cells. Date are shown in mean ± SEM. (**C**) The number of intracellular bacteria in RAW 264.7 cell after invasion by VNP20009 and its mutants. Date are shown in mean ± SEM. (**D**) Replication rates of VNP20009 and its mutants within RAW 264.7 cell. Date are shown in mean ± SEM, **P < 0.01.

Because the genes *slyA*, *STM3120* and *htrA* were reported to play vital roles in macrophage survival, each of the mutants was characterized using a gentamicin protection assay. As shown in Figure 1C, the uptake of bacteria was not impaired in the mutant groups compared with that in the VNP20009 group. However, in contrast to VNP20009, the replication rates of all mutants were decreased over five hours. VNP20009 displayed a 2.5- to 10- fold increase in replication rate in macrophages at five hour post infection compared with the mutant groups (Figure 1D).

These mutants were then characterized by probing their growth curve *in vitro*, and the growth of mutants in LB showed no defects (Figure 1A). In addition, both the mutants and VNP20009 were able to infect B16F10 cells, and there were no significant differences between the mutants and VNP20009 with respect to replication rates (Figure 1B). Collectively, these results suggested that the genes *slyA*, *STM3120* and *htrA* were not required for VNP20009 replication in melanoma cells, but were vital for VNP20009 to survive within macrophages.

### The biodistribution of the mutants in tumor bearing mice

**Figure 2:**
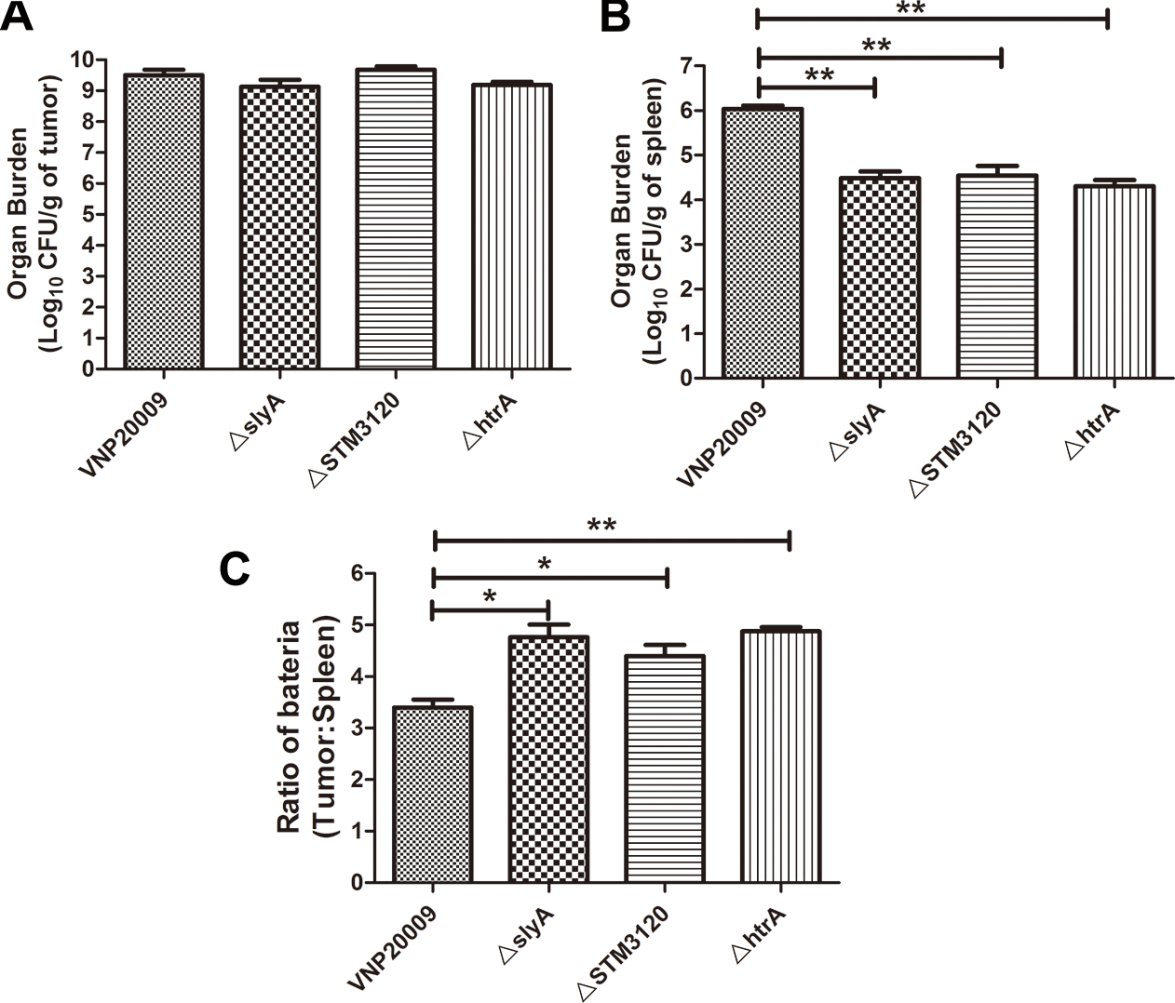
Biodistribution of VNP20009 and its mutants in tumor-bearing mice VNP20009 and its mutants were intraperitoneally inoculated into the tumor-bearing mice. The bacterial titer in spleen and tumor were determined on the third day post infection. (**A**) Bacterial burden of Δ*slyA*, Δ*STM3120* and Δ*htrA* mutants in tumor compared with VNP20009. Date are shown in mean ± SEM. (**B**) Bacterial burden of Δ*slyA*, Δ*STM3120* and Δ*htrA* mutants in spleen compared with VNP20009. Date are shown in mean ± SEM, **P < 0.01. (**C**) Tumor/spleen ratio of VNP20009 and the mutants in the tumor bearing mice. Date are shown in mean ± SEM, *P < 0.05, **P < 0.01.

To determine the biodistribution of the mutants, 1 × 10^5^ colony forming unit (CFU) *S. enterica* were inoculated into tumor-bearing mice when the tumor size reached 150 mm^3^. VNP20009 and all of the mutants targeted the tumor at similar levels, reaching above 10^9^ CFU/g in tumor 3 days post infection (*P* > 0.05) (Figure 2A). However, the bacterial loads of the mutants were significantly lower than that of VNP20009 in spleen, which is the main target of bacteria (*P* < 0.05) (Figure 2B). Additional calculations were performed to determine the ratio between the bacterial loads in tumor and spleen. The ratios of the Δ*slyA*, Δ*STM3120* and Δ*htrA* mutants were ten times higher than that of VNP20009 (Figure 2C). Overall, Δ*slyA*, Δ*STM3120* and Δ*htrA* displayed greater therapeutic potentials, as the mutants showed reduced bacterial accumulation in normal tissues and unchanged tumor fitness.

### The anticancer capacities of the mutants

**Figure 3:**
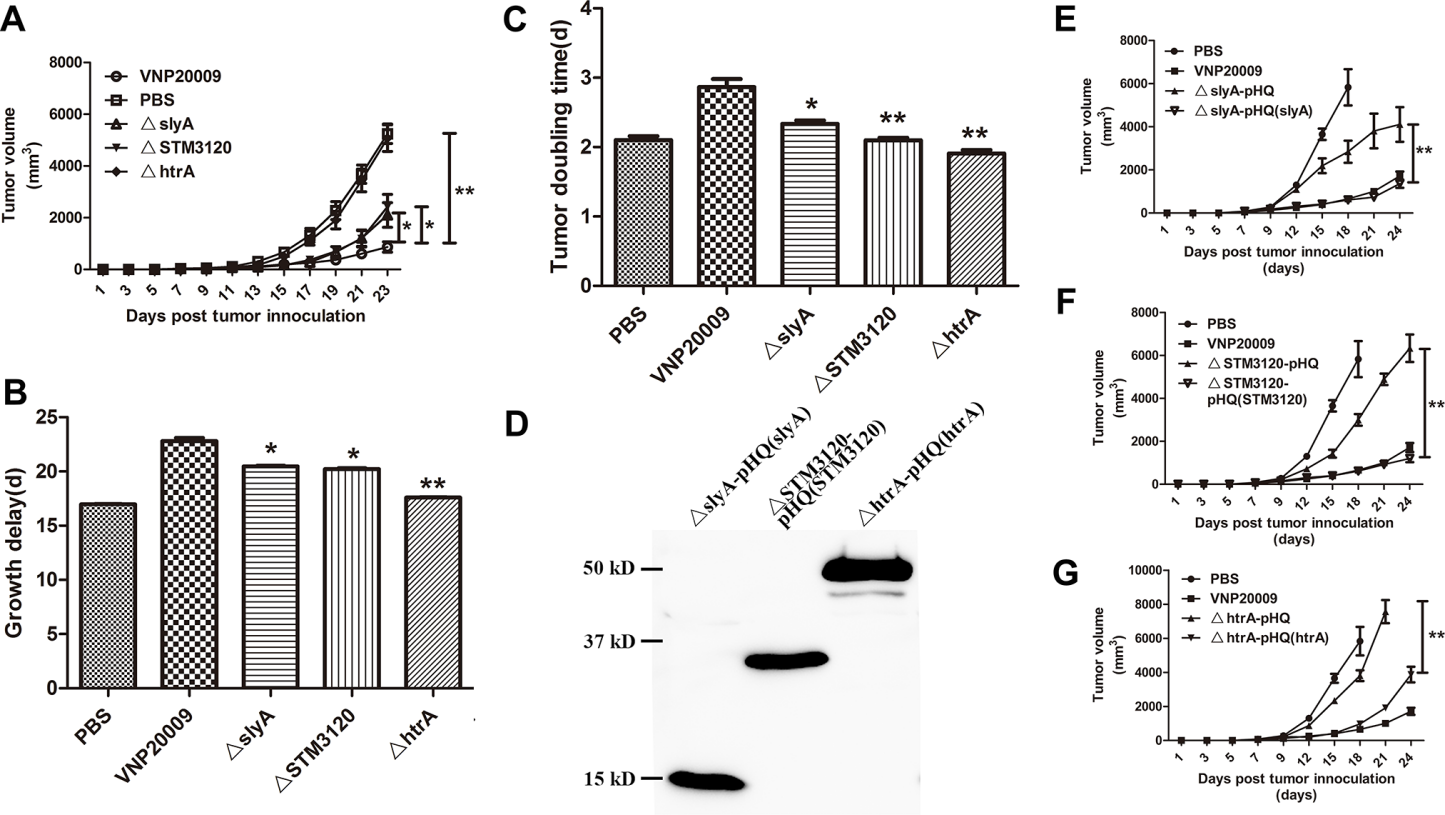
Antitumor effects of VNP20009 and its mutants *S. enterica* (1 × 10^5^ CFU/mouse) were injected intraperitoneally into the tumor-bearing mice when the tumor size reached 150 mm^3^. Tumor volumes of different groups were compared. (**A**) Tumor volumes of the mice treated with Δ*slyA*, Δ*STM3120* and Δ*htrA* compared with that treated with VNP20009. Date are shown in mean ± SEM, *P < 0.05, **P < 0.01. (**B**) Tumor growth delay of the mice treated with Δ*slyA*, Δ*STM3120* and Δ*htrA* versus VNP20009. Date are shown in mean ± SEM, *P < 0.05, **P < 0.01. (**C**) Tumor doubling time of the mice treated with Δ*slyA*, Δ*STM3120* and Δ*htrA* compared with that with VNP20009. Date are shown in mean ± SEM, *P < 0.05, **P < 0.01. (**D**) FLAG expressions from the mutants carrying pHQ003-F-slyA, pHQ003-F-STM3120 and pHQ003-F-htrA was detected by western blotting. (**E**) Tumor volumes of the mice treated with Δ*slyA*-pHQ(slyA) compared with that treated with Δ*slyA*-pHQ. Date are shown in mean ± SEM, **P < 0.01. (**F**) Tumor volumes of the mice treated with Δ*STM3120*-pHQ(STM3120) compared with that treated with Δ*STM3120*-pHQ. Date are shown in mean ± SEM, **P < 0.01. (**G**) Tumor volumes of the mice treated with Δ*htrA*-pHQ(htrA) compared with that treated with Δ*htrA*-pHQ. Date are shown in mean ± SEM, **P < 0.01.

Because the mutants displayed a higher tumor to spleen ratio than VNP20009, their anticancer activities were further investigated. The tumor suppression capacities of the mutants were partially or completely eliminated (*P* < 0.05) (Figure 3). On the 9^th^ day of infection, the tumor volumes of mice treated with VNP20009 were significantly lower than those in the PBS group (Figure 3A). Tumor doubling time and tumor growth delay were significantly increased in the mice treated with VNP20009 than those in the PBS group (*P* < 0.05) (Figure 3B and 3C). However, the mice infected with Δ*slyA* and Δ*STM3120* showed only a partial tumor growth delay compared with those infected with VNP20009 (*P* < 0.05). More importantly, Δ*htrA* failed to suppress tumor growth. The tumor doubling time was remarkably decreased from 2.69 days (CI, 2.78 d to 2.51 d) in the VNP20009 group to 2.4 days (CI, 2.52 d to 2.28 d) in the Δ*htrA* group (Figure 3C). The tumor growth delay was significantly decreased from 23.67 days (CI, 22.99 d to 24.47 d) in the VNP20009 group to 17.69 days (CI, 17.59 d to 17.70 d) in the Δ*htrA* group (Figure 3B).

In order to definitively link the genes *slyA*, *STM3120* and *htrA* to the diminished antitumor activities, the anticancer activities of Δ*slyA*, Δ*STM3120* and Δ*htrA* mutants were studied after complementation. Intact copies of the genes were cloned into pHQ-003-F, and the complementing plasmids were transformed into the corresponding mutants, respectively. After confirming the proteins were precisely expressed, we next assessed their antitumor properties (Figure 3D). As shown in Figure 3E, 3F and 3G, the anticancer capacities of the mutants were partially or completely reversed when the mutants were complemented with a copy of the corresponding gene. The presence of the vector alone did not improve the anticancer ability. These results indicated that the genes *slyA*, *STM3120* and *htrA* are vital for the anticancer ability of VNP20009.

### The analysis of the tumor immune-environment in mice

**Figure 4:**
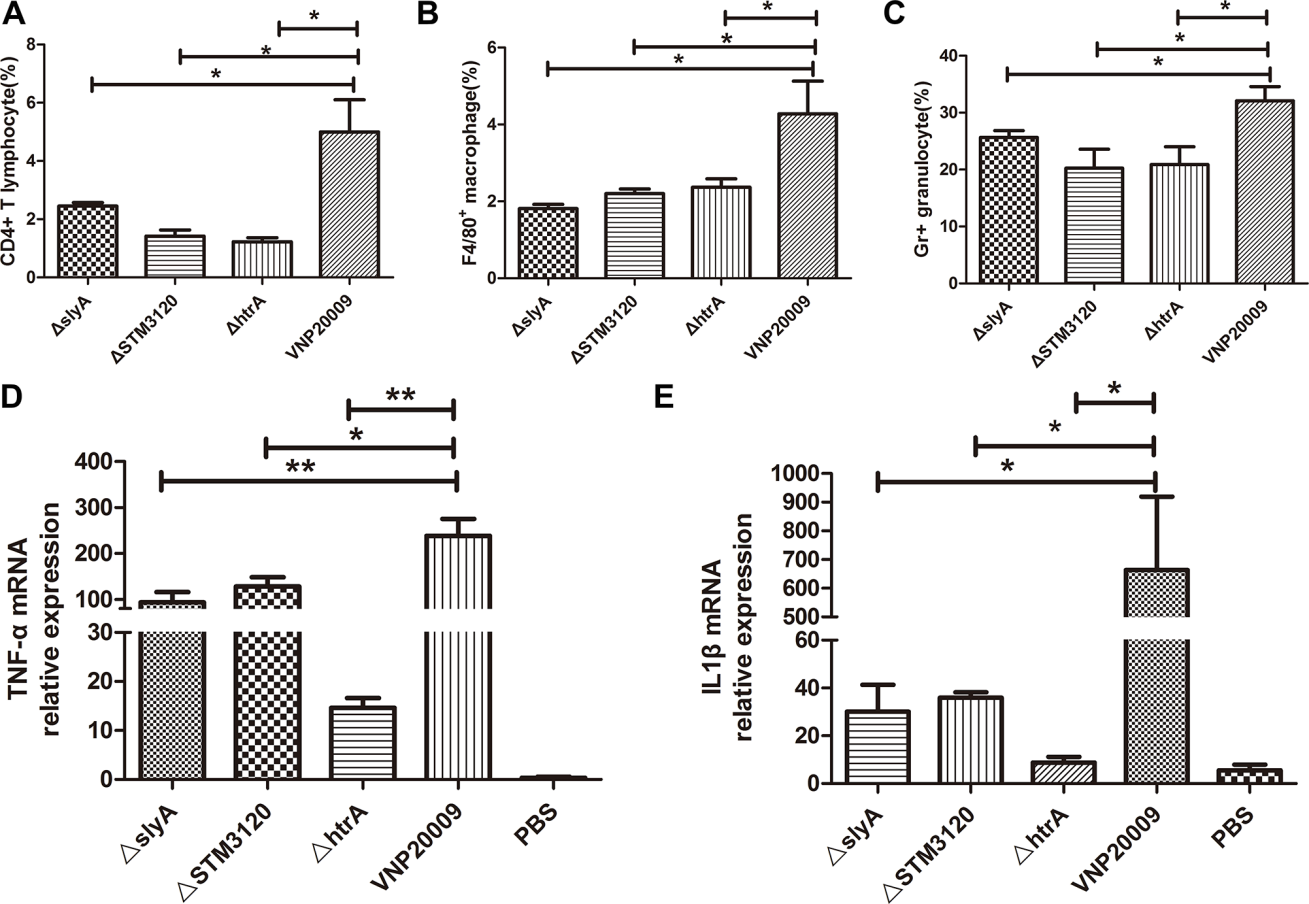
Subpopulation of immune cells and expression of TNF-α and IL-1β in the tumor VNP20009 and its mutants (1 × 10^5^ CFU/mouse) were injected intraperitoneally into the tumor-bearing mice. The immune cells in the tumor were determined on the third day post infection. (**A**) CD4^+^ T cells in the tumors of mice treated with Δ*slyA*, Δ*STM3120*, Δ*htrA* and VNP20009. Date are shown in mean ± SEM, *P < 0.05 for VNP20009 versus individual mutant. (**B)** Macrophages in the tumors of mice treated with Δ*slyA*, Δ*STM3120*, Δ*htrA* and VNP20009. Date are shown in mean ± SEM, *P < 0.05 for VNP20009 versus individual mutant. (**C**) Granulocytes in the tumors of mice treated with Δ*slyA*, Δ*STM3120*, ΔhtrA and VNP20009. Date are shown in mean ± SEM, *P < 0.05 for VNP20009 versus individual mutant. (**D**) TNF-α mRNA expression levels in the tumors colonized by Δ*slyA*, Δ*STM3120*, Δ*htrA* and VNP20009. Date are shown in mean ± SEM, *P < 0.05, **P < 0.01 for VNP20009 versus individual mutant. (**E**) IL-1β mRNA expression levels in the tumors colonized by Δ*slyA*, Δ*STM3120*, Δ*htrA* and VNP20009. Date are shown in mean ± SEM, *P < 0.05 for VNP20009 versus individual mutant.

The anti-cancer ability of *S. enterica* relies on its ability to recruit immune cells into the tumor microenvironment [19]. Therefore, whether the mutants can promote proper immune responses in tumor microenvironment remains unclear. We analyzed CD4^+^ T cells, macrophages and granulocytes in tumor, which are reported to be responsible for the anticancer ability of *S. enterica* [19–21]. The results indicated that in contrast to the mutants, VNP20009 facilitated the migration of CD4^+^ T cells, macrophages and granulocytes into the tumor (Figure 4A, 4B, 4C). The Δ*htrA* mutant, which completely lost its tumor suppressive capacity, induced 1.2% of CD4^+^ T cells on the 3^rd^ day post infection. This level was only one fifth of that induced by VNP20009 (*P* < 0.05) (Figure 4A). The macrophages and granulocytes in the tumor of Δ*htrA* group were decreased by more than 40% compared with that of VNP20009 group (*P* < 0.05) (Figure 4B, 4C). In addition, the Δ*slyA* and Δ*STM3120* treatments led a significant reduction of immune cells compared with the VNP20009 treatment (*P* < 0.05) (Figure 4A, 4B, 4C). Generally, the ability of the mutants to recruit immune cells into the tumor was reduced.

Previous studies showed that TNF-α and IL-1β are essential for bacterial cancer therapy. Thus, we further analyzed the expression of TNF-α and IL-1β in the tumors of bacteria-treated mice. The results showed that the mRNA levels of TNF-α and IL-1β in the mutant groups were significantly down-regulated compared with those in the VNP20009 group (*P* < 0.05) (Figure 4D, 4E). The expression of TNF-α in the VNP20009 group was increased 238-fold compared with that in the PBS control. However, the mice treated with Δ*slyA*, Δ*STM3120* and Δ*htrA* mutants exhibited only 93-, 128- and 28- fold increase in TNF-α expression, respectively, compared with the mice treated with PBS (Figure 4D). Although IL-1β was significantly increased after bacterial therapy, the expression of IL-1β in the VNP20009 group was increased greater than 18- fold compared with the mutant groups (*P* < 0.05) (Figure 4E). These results indicated that the reduced anticancer activities of the mutants, which were deleted the genes *slyA*, *STM3120* and *htrA* in the VNP20009 background, might be ascribed to the inability to activate immune-mediated antitumor responses.

## DISCUSSION

VNP20009 is a live anti-cancer agent and has some unique properties to make it better than traditional methods, including chemotherapy and radiation. For example, VNP20009 can specifically target tumors, sense the local environment and respond to external signals and can be engineered at genetic levels [1]. However, it failed in phase I clinical trials because of no antitumor effects [22]. Thus, tremendous efforts, including whole genome sequencing, were performed to improve the safety profile and tumor targeting ability [23]. However, the underlying mechanism of the anticancer capacity of VNP20009 still remains unclear. In this study, we knocked out the genes *slyA*, *STM3120* and *htrA*, which are responsible for bacterial survival within macrophages, in the background of VNP20009 to study the relationship between bacterial intramacrophage survival ability and antitumor capacity. We found that all the mutants partially or completely lost their antitumor capacities, although they preferentially targeted and colonized tumor. The result indicates that in contrast to the former assumption, the bacterial burden in tumor does not play a decisive role in bacterial anticancer ability, which is consistent with previous observations [24, 25].

To establish a systematic infection, *S. enterica* must survive and replicate within the host macrophages where it is exposed to poor nutrients and microbicidal environments [26]. Production of reactive oxygen species (ROS) and nitric oxide is an important feature of intracellular bactericidal activities, which can be prevented by expressing *slyA*, *STM3120* and *htrA* genes. HtrA possesses protease and chaperone activities, which are temperature dependent *in vitro* [27, 28]. The chaperone activity of HtrA predominates at low temperatures, whereas protease activity is present at high temperatures. In the present study, the growth rate of Δ*htrA in vitro* was not impaired at 37°C, although the growth of Δ*htrA* was reported to be inhibited at 46°C or higher [27]. In addition, Mutunga et al. found that Δ*htrA* was attenuated because of increased sensitivity to oxidative stress [29]. Thus, Δ*htrA* mutant was evaluated as a live vaccine and used for delivering recombinant antigens, such as CVD 908-htrA [30]. SlyA, a member of the MarR family of transcription factors, is a transcription factor that regulates a large number of genes, including *S. enterica* pathogenicity island (SPI)-2 [31, 32]. Because the genes within SPI-2 were reported to be involved in *S. enterica* virulence by preventing killing by mechanisms involving oxidative mechanism and because SPI-2 is required for *S. enterica* survival within macrophages, Δ*slyA* is sensitive to oxidative products of the respiratory burst [33, 34]. STM3120 is a predicted *citE* (citrate lyase) homolog and is important during systemic infection in BALB/c mice. Santiviago et al. suggested that STM3120 could decrease the level of nitric oxide in macrophages [35]. Consistent with these findings, our results revealed that the deletion of the genes *slyA, STM3120* and *htrA* in the background of VNP20009 made the mutants more vulnerable in macrophages, although their growth rates *in vitro* and replications in B16F10 cells were not impaired.

The results showed that Δ*slyA*, Δ*STM3120* and Δ*htrA* exhibited the highest tumor to spleen ratio, indicating that the tumor speci ficity of *S. enterica* was closely related to survival capacity within macrophages. When *S. enterica* enters the blood, the immune system is activated to deplete bacteria from the circulation system and macrophage is probably the most important. Given that *S. enterica* is rapidly subjected to phagocytosis by macrophages, only those bacteria capable of replicating within macrophages can cause a systematic infection. Thus, *S. enterica* that is incapable of replicating intracellularly is highly attenuated. In the present study, Δ*slyA*, Δ*STM3120* and Δ*htrA* were efficiently cleared by macrophages, which could explain the low bacterial burden in spleen.

By contrast, *S. enterica* in tumors are protected from the immune system, and all the mutants accumulated in the tumor to the same extent as VNP20009. Although the process of bacterial tumor targeting is not entirely clear, macrophage was reported not to be a transporter of bacteria to tumor, and depleting macrophages did not affect tumor colonization [36]. Our results further confirmed this finding because the mutants with reduced survival within macrophages reached the same accumulation level in tumors as VNP20009. In addition, *S. enterica* was found both intracellular and extracellular in the tumor, where immune surveillance fails to function [36]. Three days post infection, when the bacterial titer reached the highest level in the tumor, all the mutants reached the same accumulation level in tumor as VNP20009 because the growth and replication rates of the mutants within melanoma cells were not impaired [37]. These results further reinforce that the low accumulation of mutant strains in spleen is due to impaired survival ability in macrophages. Although further investigations are necessary, the results showed that the bacterial tumor selectivity depends on macrophage-mediated clearance from the systemic organs and persistence of bacteria in the tumor microenvironment, which also partially explain Hoffman's discovery, which showed mutants in *STM3120* and *htrA* displayed reduced fitness in normal tissue and unchanged fitness in tumor [13].

Given that Δ*slyA*, Δ*STM3120* and Δ*htrA* exhibited therapeutic potentials with reduced colonization in normal organs and unchanged colonization in tumors, they were thus validated for their anticancer activities. All of the mutants partially or completely lost their anticancer abilities. When the mutants were complemented with respective genes, their antitumor activities were partially or completely restored. In addition, the immune cells in the tumors of mice infected with the mutants were significantly decreased. Previous studies showed that the anti-tumor effects of *S. enterica* depend on the functional role of the MyD88-TLR pathway, and *S. enterica* enhances anti-tumor immunity by inhibiting tumor indoleamine 2, 3-dioxygenase 1 expression and inducing gap junctions in tumors [20, 38, 39]. Our results indicated the genes *slyA*, *STM3120* and *htrA*, which are indispensable for the anticancer activity of VNP20009, are required for VNP20009 to induce the immune cells and exert an anti-tumor effect.

Furthermore, TNF-α and IL-1β are important for the tumor suppressive effects of *S. enterica*. Kim et al. found that the co-administration of recombinant TNF-α could prolong the tumor-suppressive effects of ΔppGpp *Salmonella* [25]. In our study, TNF-α was significantly decreased in the mice treated with the mutants. In addition, the mutants with impaired anticancer ability induced far less IL-1β than VNP20009. A recent study showed that the impaired anticancer ability of MG1655 (LPS-competent *E.coli*), which displayed similar tumor targeting capacity as *S. enterica*, is due to reduced IL-1β expression [25]. The result may also explain why the *S. enterica* mutants with unchanged tumor targeting ability failed to suppress tumor growth.

In conclusion, we created Δ*slyA*, Δ*STM3120* and Δ*htrA* mutants that had similar tumor targeting abilities, but reduced anticancer capacities compared to VNP20009. We found that the survival ability within macrophages can increase the bacterial burden in normal organs without affecting the bacterial tumor targeting ability. In addition to this finding, immune cells and cytokines within tumors, rather than the bacterial burden in tumors, are responsible for the anticancer activity of VNP20009. The current study is the first to show the relationship between macrophage intracellular survival and anticancer activity and thus provides novel insights into bacterial cancer therapy.

## MATERIALS AND METHODS

### Bacterial strains, cell lines, and mice

VNP20009 is a purine auxotrophy, which deletes *msbB* from *S. enterica* ATCC 14028S [8]. All the mutants, namely Δ*slyA*, Δ*STM3120* and Δ*htrA* with a deletion of *slyA*, *STM3120* or *htrA* in VNP20009 were gifts from Prof. Shi's lab. All the strains of *S. enterica* were cultured in Luria Broth (LB) or on agar plates using standard procedures. B16F10 melanoma cells were obtained from American Type Culture Collection (ATCC, USA) and were cultured in 5% CO_2_ in a humidified atmosphere in Dulbecco's modified Eagle's media (DMEM) supplemented with 10% fetal bovine serum (FBS). Six-week-old female C57BL/6 mice were purchased from the Comparative Medicine Center of Yangzhou University and maintained in pathogen-free conditions for one week before treatments.

### Quantification of *S. enterica* in tumor

Briefly, 1 × 10^5^ B16F10 cells were subcutaneously inoculated into the mid-right flank of C57BL/6 mice. When the tumor size reached 150 mm^3^, mice were randomly divided into VNP20009 group, Δ*slyA* group, Δ*STM3120* group and Δ*htrA* group, with each group containing of at least 4 mice. *S. enterica* was grown overnight in LB and was diluted at a ratio of 1:100 into fresh LB. When optical density (OD) at 600_nm_ was between 0.7 and 1.0, *S. enterica* with 1 × 10^8^ CFU were obtained through centrifuging and suspended in phosphate buffered saline (PBS). *S. enterica* with 1 × 10^5^ CFU were obtained by a serial dilution and intraperitoneally injected into tumor-bearing mice. The mice were sacrificed 3 days after *S. enterica* injection. Spleen and tumor were homogenized in 2 ml of PBS to release *S. enterica* and then plated on LB agar by a serial dilution. After 16 h of culture at 37°C, the titer of *S. enterica* was determined by counting colonies and dividing them by the weight of the tissue (CFU/g) [40].

### Anti-tumor assay *in vivo*

1 × 10^5^ B16F10 cells were subcutaneously inoculated into the mid-right flank of C57BL/6 mice. When the tumor size reached 150 mm^3^, tumor bearing mice were randomly divided into groups, with each group containing 8 mice. For comparing the antitumor activities of the mutants and VNP20009, 1 × 10^5^ CFU *S. enterica* were intraperitoneally inoculated into the tumor-bearing mice. For complementation assay, the mutants carrying corresponding plasmids and the mutants were intraperitoneally inoculated into the tumor-bearing mice, respectively. The length and width of the tumors were measured every two days by using a Vernier caliper (Mytutoyo Co., Japan) across two perpendicular diameters. Tumor volume was calculated using the following formula: tumor volume = length × width^2^ × 0.52.

### Flow cytometry

As mentioned above, tumor bearing mice were randomly divided into 5 groups, with each group containing 4 mice. When the tumor size reached 150 mm^3^, 1 × 10^5^ *S. enterica* were intraperitoneally inoculated into the tumor-bearing mice. The tumor-bearing mice were sacrificed on the third day. According to Bayne and Vonderheide's work, tumors were collected post infection for subpopulation analysis of immune cells [41]. The tumor was digested by collagenase IV. A single cell suspension, which was obtained using a 40-μm cell falcon, was incubated in red blood cell lysis buffer for three to five minutes to exclude red blood cells. The cells were then washed twice in DMEM medium. To determine the percentage of immune cells in the tumor, 10,000 cells suspended in 100 μl PBS containing 10% fetal calf serum (FCS) were stained for 30 min at 4°C by using appropriate isotype controls and phycoerythrin-conjugated anti-CD4, anti-F4/80, FITC-conjugated anti-Gr (BD Biosciences). After washing in PBS, the cells were analyzed on a FACSCalibur using CELLQUEST software (BD Biosciences).

### Bacterial invasion and amplification assay *in vitro*

The day before infection, 1 × 10^6^ B16F10 or RAW264.7 cells were seeded into 12-well plate with 500 μl appropriate cell culture medium. The cells were incubated at 37°C in 5% CO_2_. The cell culture medium was replaced with fresh medium one hour prior to infection. 1 × 10^7^ CFU bacteria were added into each well. After infection, the plates were centrifuged at 250 g for 5 minutes in order to bring cells and bacteria in contact and initiate infection. The plates were then incubated for one hour at 37°C in 5% CO_2_. Gentamicin (100 μg/ml) was added to the plates to kill extracellular bacteria one hour after infection. The gentamicin concentration was reduced to 10 μg/ml two hours later. At designated time points, cells were washed with PBS and lysed by 0.1% Triton X-100. Bacteria were released and plated onto LB agar plates by a serial dilution. CFUs on the plates were counted after overnight incubation at 37°C.

### Real-time quantitative PCR assay

As mentioned above, tumor bearing mice were randomly divided into 5 groups, with each group containing 3 mice. Briefly, 1 × 10^5^ *S. enterica* were intraperitoneally inoculated into the tumor-bearing mice. 7 days post infection, the mice were sacrificed and total RNA was isolated from tumor tissues with TRIzol reagent (Invitrogen). cDNA was synthesized using ReverTra Ace^®^ qPCR RT Kit (Toyobo). Real-time quantitative PCR was performed using AceQ qPCR SYBR Green Master Mix (Vazyme) to determine TNF-α and IL-1β expression. β-actin was used as a normalizing control.

### Construction of complementing plasmids

The genes *slyA*, *STM3120* and *htrA* were amplified for complementation using the primer sets as listed in Table 1. The resulting PCR products were purified using agarose gel electrophoresis and extracted from the gel using the gel extraction kit. The purified PCR products were inserted into pHQ003-F, a derivative of pET28a vector by ClonExpress II One Step Cloning Kit (Vazyme). The expression of the gene is controlled by a constitutive J23100 promoter and the FLAG tag is attached to the C terminus of complement protein. *E.coli* DH5α was transformed with the complement plasmid, and the positive clones were selected on LB plates containing Kanamycin. Plasmids bearing the correct insert were electroporated into the mutants, namely Δ*slyA*-pHQ(slyA), Δ*STM3120*-pHQ(STM3120) and Δ*htrA*-pHQ(htrA), respectively. Plasmid-bearing mutants were selected on LB Kna plates.

**Table 1:** Primer sequence used to construct complementing plasmids

	Name	Sequence (5′–3′)
slyA	slyA-F	gaggagaaatactag*ttggaatcgccactaggttc*
	slyA-R	tttgtagtcaagctt*atcgtgagagtgcaattcca*
STM3120	STM3120-F	gaggagaaatactag*atgcccgatatttctcatac*
	STM3120-R	tttgtagtcaagctt*ggaaaagatccctgcccggg*
htrA	htrA-F	gaggagaaatactag*atgaaaaaaaccacattagc*
	htrA-R	tttgtagtcaagctt*ctgcatcagcaaataaatag*

### Western bolt analysis

Δ*slyA*-pHQ(slyA), Δ*STM3120*-pHQ(STM3120) and Δ*htrA*-pHQ(htrA) were grown overnight in LB broth containing Kanamycin. Bacterial pellets were boiled in SDS loading buffer for 15 min. Blots were probed using monoclonal mouse antibody (F1804) against FLAG (Sigma).

### Statistical analysis

The results are presented as mean ± SEM using Graphpad software. Statistical significance for comparisons among two was analyzed using the student t test by SPSS. The level of significance was set at *P* < 0.05.
